# A Novel Method Based on GAN Using a Segmentation Module for Oligodendroglioma Pathological Image Generation

**DOI:** 10.3390/s22103960

**Published:** 2022-05-23

**Authors:** Juwon Kweon, Jisang Yoo, Seungjong Kim, Jaesik Won, Soonchul Kwon

**Affiliations:** 1 Department of Electronic Engineering, Kwangwoon University, Seoul 01897, Korea; 02kjw0203@kw.ac.kr (J.K.); jsyoo@kw.ac.kr (J.Y.); 2 Molpaxbio, Daejeon 34047, Korea; jong.kim@molpax.com (S.K.); jaesik.won@molpax.com (J.W.); 3 Graduate School of Smart Convergence, Kwangwoon University, Seoul 01897, Korea

**Keywords:** generative adversarial networks, pathology image synthesis, digital pathology

## Abstract

Digital pathology analysis using deep learning has been the subject of several studies. As with other medical data, pathological data are not easily obtained. Because deep learning-based image analysis requires large amounts of data, augmentation techniques are used to increase the size of pathological datasets. This study proposes a novel method for synthesizing brain tumor pathology data using a generative model. For image synthesis, we used embedding features extracted from a segmentation module in a general generative model. We also introduce a simple solution for training a segmentation model in an environment in which the masked label of the training dataset is not supplied. As a result of this experiment, the proposed method did not make great progress in quantitative metrics but showed improved results in the confusion rate of more than 70 subjects and the quality of the visual output.

## 1. Introduction

Pathology is a branch of medicine in which the morphological changes that occur in tissue, organs, and cells are microscopically observed to investigate the causes of disease. It provides important information for making final diagnostic decisions. Latterly, pathology has used electron microscopes to magnify brain tumor, muscle, or other tissue by thousands to tens of thousands of times to observe minute morphological changes. This digitization is called digital pathology, and accordingly, digital pathology data analysis research based on deep learning is being studied using a large amount of data. In recent years, in the field of pathology, deep learning has been used to analyze and predict data and has achieved state-of-the-art performance. Through various deep learning-based algorithms, digital pathology mainly designs classifiers for diagnosing pathologist-level diseases and to assist in human decision-making processes. It has also been used for detecting or segmenting specific parts, such as nuclei, cells, and blood vessels, in slide images, normalizing H&E stained images and predicting patients’ life expectancies.

For training, deep learning networks generally require tens of thousands to millions of samples in the datasets. However, collecting data from medical imaging remains difficult, and the data imbalances between classes are a problem. Data augmentation techniques and methods of synthesizing data have been actively researched to solve this problem. General data augmentation techniques, such as x-flips, y-flips, $90^{\circ }$ rotations, color transforms, brightness control, noise addition, etc., have been used to create data to improve and generalize the performance of deep learning networks. In addition to the common data augmentation techniques, machine learning algorithms are also widely used. Representatively, there is the Synthetic Minority Oversampling TEchnique (SMOTE) [1] that uses the k-nearest neighbors (K-NN) algorithm. He et al. and Han et al. oversampled the data using both the adaptive synthetic sampling (ADASYN) [2], and Borderline-SMOTE algorithms are based on SMOTE [3]. Deep learning algorithms also demonstrated good performance in data synthesis. Generative adversarial networks (GANs) [4] have actively studied a representative generative model for data synthesis and have produced several unanticipated applications.

In this paper, we present a method to generate oligodendroglioma (ODG) pathology images from latent space using the GAN for generating realistic data. Unlike many previous GAN algorithms, we propose the following: a method of applying a simple feature concatenation that assists in training the generator. To apply the feature concatenation technique requires a segmentation module consisting of symmetrically designed segmentation and reconstruction networks based on the U-net architecture [5]. The segmentation module consists of the segmentation network that segments important features in cellular tissue, such as nuclei and glands, and a reconstruction network that helps improve the segmentation accuracy by reconstructing the original image from the masked image, which is the result of segmentation. Similar to most segmentation tasks, a training dataset consisting of pairs of the original and labeled data are often used for supervised learning. The Cancer Genome Atlas (TCGA) LGG dataset we used does not have labeled data; therefore, for training, we provided a guideline dataset using k-means clustering [6] to our segmentation model and changed from unsupervised training to a simple method. The generator produces realistic ODG images from the random latent vector *z* by utilizing the features that are used in the reconstruction stage of the segmentation module. Our simple method of guiding the generator by concatenating the embedding feature maps of the reconstruction network created better quality images for the TCGA data than the existing method.

Works related to this study are described in Section 2. We propose our method in Section 3 and describe the qualitative and quantitative experimentation results in Section 4. Finally, we include the discussion and conclusion in Section 5 and Section 6, respectively.

## 2. Related Work

### 2.1. Oligodendroglioma

Oligodendroglioma (ODG) are rare tumors that infiltrate a wide range but mainly occur in adult cerebral hemispheres’ white matter, and they consist of cells resembling oligodendrocytes or oligodendroglia [7,8]. Histologically, ODG is characterized by round nuclei with a rounded cytoplasmic surround. Because of this perinuclear halo characteristic, it is often referred to as the “fried egg appearance”. In addition, “uniformly round to oval-shaped cells”, “uniform nuclei”, the “chicken-wire capillary network”, and “calcification” are frequently found characteristics [7,8].

According to statistics from the Central Brain Tumor Registry of the United States (CBTRUS) from 2011 to 2015, the incidence of ODG, including anaplastic oligodendroglioma, was 3.57 to per 100,000 [9]. Kleihoues reports that ODG is a brain tumor that accounts for about 5–18% of all intracranial gliomas [7]. Suh et al. reported that it accounted for approximately 3.4% of the total central nervous system gliomas [10].

### 2.2. Histology Image Synthesis

By training deep learning networks, data augmentation techniques improve the performance of deep learning models by increasing the number of training samples. Various techniques are used when the training data are insufficient. This is essential for medical data that are severely imbalanced in each class. Using methods such as x-flips, y-flips, $90^{\circ }$ rotations, color transforms, brightness control, and noise addition increases the generalization performance of deep learning models by creating a large quantity of relevant data. Data augmentation methods may yield desired performance depending on the task, but sometimes, it degrades the network’s performance. For example, for a human face dataset, such as the CelebA [11] and FFHQ [12] datasets, if color transformations are strongly applied, samples that are highly unlikely to exist can be added as the training data. Therefore, rather than simply sampling meaningless data, meaningful augmentation techniques must be applied such as increasing the number of data points in a minority layer that has insufficient data. The SMOTE finds the nearest neighbor within the minority class and multiplies the difference between samples using a random value of between 0 and 1 to create new data samples. Consequently, it matches the quantity of data between classes [1]. He et al. proposed ADASYN, which improves SMOTE. This work applies the concept of assigning weights according to the density of the majority class when selecting a small-class sample [2]. In addition, Han et al. proposed Borderline-SMOTE, which synthesizes new data samples by applying SMOTE based on the boundary between the minority class sample set and the majority class sample set [3].

### 2.3. Segmentation

Several studies have been conducted on pixel-accurate segmentation for digital pathology. In particular, it is common to use U-Net [5] in medical imaging tasks, such as data segmentation, reconstruction, and pre-processing. U-Net is a U-shaped architecture-based model in a fully convolutional network [13]. This model can execute end-to-end learning using small datasets, resulting in efficient segmentation for biomedical imaging. Two paths, one contracting and the other expanding, function as the encoder and decoder, respectively. The two paths are connected via skip connection. This structure ensures successful segmentation results by preserving local information. In digital pathology, previous studies have used the U-Net structure to segment various cancers, such as in breast [14,15,16,17,18], colon [19,20,21,22,23], lung [24,25], and prostate tissue [26,27]. Furthermore, the structure is also applied to localized tissues such as the nuclei [24,28,29,30,31,32], cells [33,34] and glands [19,20,21,22,23], which exhibit major pathological characteristics. Currently, from the structural perspective, some relevant studies have used network-modified U-Net structures, such as Dual-U-Net [32], U2-Net [35] and R2U-Net [31]. We followed the fundamental structure of previous segmentation studies and used them to stabilize the training and contribute to improving performance by combining it with GANs.

### 2.4. GANs in Digital Pathology

Generative adversarial networks (GANs) introduced by Goodfellow are deep learning-based generative models. A GAN consists of a generator (G) that maps random noise *z* to the target data distribution and a discriminator (D) that estimates the probability that the data came from the target data distribution or G. The two networks increase each other’s performance via the adversarial training process. GANs face critical problems during training. First, they are unstable during training and several studies have focused on solving this problem. Representatively, the DCGAN [36] suggests guidelines for stable training, and this structure is very effective. The WGAN mathematically proved that if the 1-Lipschitz constraint is satisfied, satisfactory convergence can be achieved using the Earth Mover Distance (EMD) and Wasserstein distance (WD) [37]. The WGAN-GP’s training stability and mode collapse problem is resolved by applying a gradient penalty to the WGAN [38]. There are studies, such as LSGAN [39], hinge loss-based GAN [40], unrolled GAN [41], MDGAN [42], and SN-GANs [43], that improve GANs from the loss-function perspective. In addition, studies such as LAPGAN [44], PGGAN [45], StyleGAN [12], EBGAN [46], SAGAN [47], and BigGAN [48] that have improved the structure of the models. PathologyGAN [49], which generates pathological data, is a network that uses BigGAN as a baseline architecture. The BigGAN is a model that stably trained GANs with a large dataset such as the ImageNet dataset [50], which consists of 14 million images and thousands of classes by applying the Spectral Normalization (SN) and Self-Attention (SA) layers to both G and D networks. PathologyGAN used Relativistic Average Discriminator [51] and succeeded in generating high-quality fidelity data with faster convergence when hinge loss is used as the GAN’s loss function. In addition, a structure that maps the latent vector *z*, the input of *G*, to w∼M(z) , through StyleGAN’s mapping network and adaptive instance normalization (AdaIN) was used. Quiros et al. used two H&E breast cancer datasets from the Netherlands Cancer Institute (NKI) and Vancouver General Hospital (VGH) cohort, and they achieved a Fréchet Inception Score (FID) of about 16.65 [49]. Deshpande et al. generated tissue images using CGAN [52] for the CRAG (Colorectal Adenocarcinoma Gland) dataset, and they combined several small tiles to create high-resolution images with realistic pathological features such as gland appearance and nuclei structure [53]. Sondre et al. generated synthetic data from random latent vectors by combining CycleGAN [54] and ACGAN (Auxiliary classifier GAN) [55] for dermoscopic skin lesion images. Sondre et al. applied image transfer via CycleGAN to a common class (nevus) to create a rare and specific melanoma class [56]. Boyd et al. [57] generated pathological data with realistic and fine details through visual field expansion method and reported a FID of approximately 21 and 37 for CAMELYON17 [58] and CRC [59] datasets.

Digital pathology uses deep learning methods primarily for classification, segmentation, or tasks that assist medical prediction. In recent years, research on the image-generation task has increased, and pathological image generation using GANs in particular has the potential for various applications.

In this study, we propose a method for generating data by applying a simple method called a ‘’embedding feature concatenation’’ to the conventional GAN architecture. We generated ODG images using the TCGA dataset, and the overall workflow was divided into two parts: the segmentation module and the generation module.

## 3. Method

We propose a method to synthesize oligodendroglioma (ODG) pathology data. Our model is based on the PGGAN, which among GAN models, achieves high performance. For generating realistic images, we trained our generator using latent vector *z* and embedding features.

**Figure 1:**
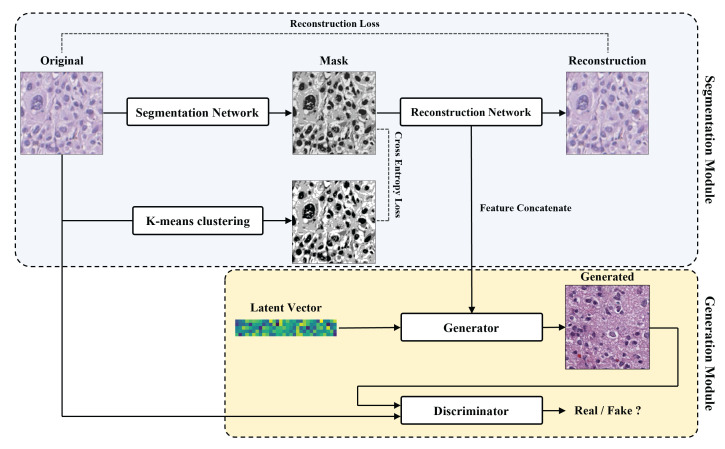
The overall flow of our proposed model. A segmentation module (light blue box), a generation module (yellow box).

As illustrated in Figure 1, our proposed method consists of two modules.

- The segmentation module creates masked images from the reference images and extracts meaningful features in the reconstruction process.
- The generation module generates pathological images from the features of the segmentation module and latent vectors by the embedding feature concatenation method.

The segmentation module consists of two networks, one creates masked images from pathological images and the other reconstructs the reference images. The ODG dataset that we used has the following problem: the inability to produce segmentation labels. Therefore, k-means clustering was used to solve this problem. The generation module was designed based on the PGGAN. The PGGAN achieves good performance in general cases but often generates poor visual quality in the pathology domain. To generate visually realistic images, we concatenate the embedding features. This method concatenates output features of the segmentation module to the corresponding scale level of generator layers. Consequently, we generated realistic, high-quality ODG data.

### 3.1. Segmentation and Reconstruction Module

In recent years, pixel-level segmentation studies have been conducted in digital pathology. Segmentation in the pathological domain distinguishes various regions such as cell nuclei, cells, and glands.

**Figure 2:**
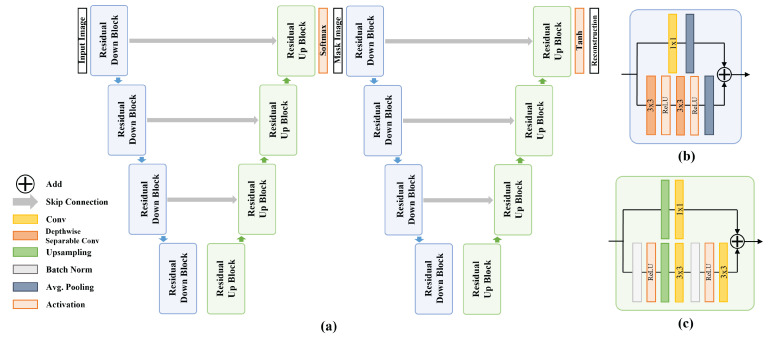
The segmentation module structure. (**a**) The structure of the segmentation network and reconstruction network. (**b**) The layer structure of the residual down-sampling block. (**c**) The layer structure of the residual up-sampling block.

Our segmentation module aims to extract meaningful embedding features from reference images. The architecture was composed of two networks, as shown in Figure 2a. The first network is a segmentation network. The network creates masked images by discriminating blood vessels, cells, nuclei, and cytoplasm from the input pathology images. The second network reconstructs the masked results obtained by the reconstruction network into original input images of the segmentation network. Subsequently, the feature outputs of the intermediate layers of the reconstruction network are concatenated to train the generator. Additionally, we observed that the segmentation accuracy improved by adding the reconstruction network.

Both networks were designed based on Res-UNet [60], which is a residual block applied to U-net. We designed networks based on both Res-UNet and the residual blocks applied to U-net. To captures the local information of the input accurately, both networks have a symmetrical structure. We configured the two networks in the same U-net architecture using four pairs of residual down-sampling blocks (RDB) and residual up-sampling blocks (RUB). Figure 2b,c show the structural layouts of the RDB and the RUB, respectively.

The RDB consists of two paths. One path connects two 3×3 depth-wise separable convolution layers and an average pooling layer. The other goes through a 1×1 convolution and an average pooling layer. Subsequently, the outputs of both paths are added. The RUB adds a path that connects an up-sampling layer and two 3×3 convolution layers and the path of an up-sampling layer and a 1×1 convolution layer.

We generated masked images using softmax in the last RUB, resulting in a segmentation network and obtained reconstructed images by using tanh in the reconstruction network.

In general, the segmentation network aims to learn the function f:X→Y , where *X* is the dataset of pathological images, *Y* is the corresponding mask label and the reconstruction network aims to learn the function g:Y→X , which reconstructs the original input data.

Most studies on segmentation tasks use a paired dataset containing data with corresponding ground truth labels. Unfortunately, many medical datasets including the TCGA dataset we used do not provide segmentation labels.

We used k-means clustering to train the segmentation module using the TCGA dataset, because k-means clustering is a simple algorithm requiring few computational costs. We labeled groups of pixels with similar colors using k-means clustering. The TCGA dataset consisted of H&E-stained pathology images, and the images were stained with similar colors for cells, nuclei, and cytoplasm. Therefore, we conducted pixel grouping of similar colors using k-means clustering and classified the cells, nuclei, and cytoplasm with relevant labels.

As shown in Figure 1, we used the mask image obtained by k-means clustering as ground truth labels and used the cross-entropy loss between the k-means clustering results and the segmentation results in the training process. Using this method, we addressed the problem of ground truth labels not being simply provided.

**Figure 3:**
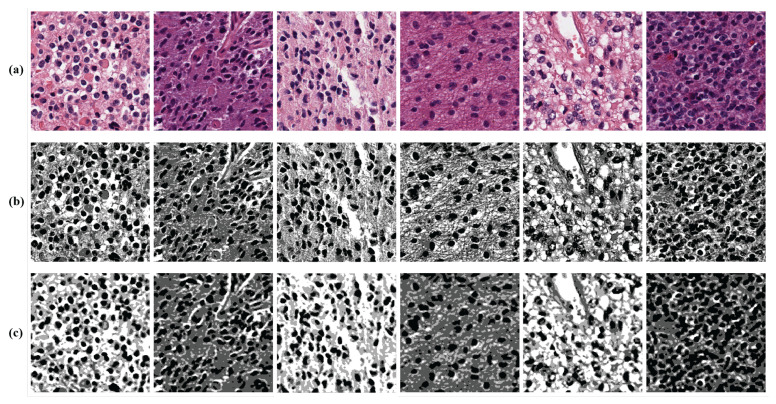
Examples of images obtained through k-means clustering and the segmentation results. (**a**) The samples of the segmentation network input. (**b**) The samples of k-means clustering results. (**c**) The samples of the mask images of the segmentation module.

The segmentation operation results in an integer value of class per each pixel via softmax. It means that the masked data have k-channels as the output of the softmax layer, and each channel value is expressed as a probability of *k* classes. For visualization and training the reconstruction network, we mapped the results to the class corresponding to the highest probability. Then, we normalized the result to a value of [0, 1]. Consequently, our segmentation network generated the gray-scale masked data with a resolution of 256×256 , as shown in Figure 3c. Figure 3a shows samples of TCGA input data, and Figure 3b shows examples of k-means clustering results.

**Figure 4:**
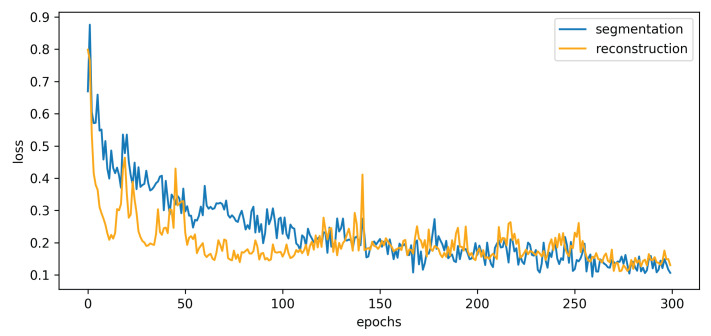
The training loss of the segmentation module.

Figure 4 shows the loss during training of the segmentation module. The two lines are the loss of the segmentation network and the reconstruction network, respectively. Additionally, the embedding features of the reconstruction network were concatenated into the layers of the generator for each scale level. Thus, the embedding features extracted from the segmentation module improve the visual performance of the baseline.

### 3.2. Concatenating Embedding Features in the Generation Module

This section describes how to concatenate embedding features into a general GAN structure. We generated ODG data by applying the proposed method based on a progressive growing GAN (PGGAN), which demonstrated stable training and high-quality image generation. Initially, the PGGAN maps the latent vector to the 4×4 low-resolution samples. In addition, this model adopts the progressive training method of stacking new layers by increasing the resolution by a power of 2. Consequently, it stably generates high-resolution images. Similarly, we progressively trained our model by the proposed method concatenating the embedding features extracted from the reconstruction network of a pre-trained segmentation module of a PGGAN-based generator. As shown in Figure 2a, the reconstruction network of the segmentation module consists of 4 RDBs that constitute the encoder and 4 RUBs that constitute the decoder. We extracted the embedding feature outputs from three blocks: the last RDB, first and second RUBs. Each feature map was concatenated according to the output scale of the generator.

Therefore, the generator uses the information from the reconstruction network and supports GAN training with the process formulated as:

(1) $$Img_{G}=G\left(z,F_{enc},F_{dec}\right)$$

where G(·) is a generator, z∼N(0,1) is a latent vector, and $F_{enc}$ and $F_{dec}$ indicate the output feature maps of the encoder and decoder of the reconstruction network, respectively.

**Figure 5:**
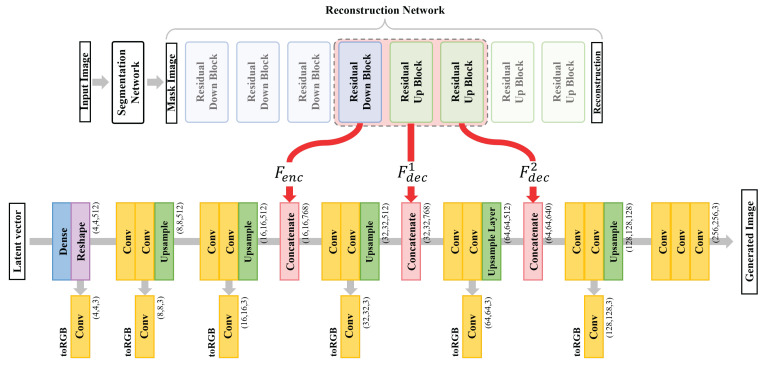
The embedding feature concatenation method and the layer structure of the generator of the generation module.

**Figure 6:**
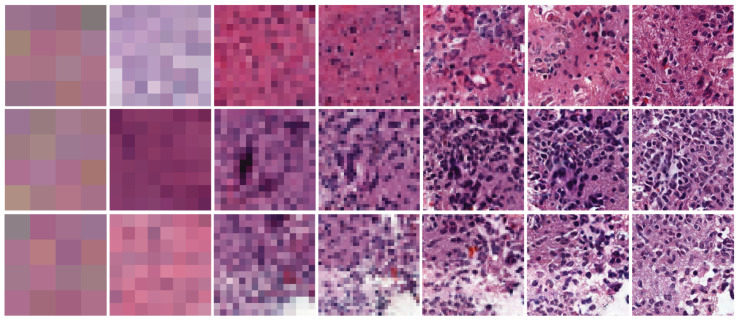
Low to high-resolution data samples were progressively generated by our model. From 4×4 (**leftmost**) to 256×256 (**rightmost**).

Figure 5 shows how to apply the embedding feature concatenation method during progressive training. Our training process also learns an initial generator that creates $Img_{init}\in R^{4\times 4}$ images from the latent vector $z\in R^{512}$ in the same manner as the PGGAN, and the new layers fade in smoothly in subsequent steps. In the intermediate step, to generate 16×16 resolution, the feature maps $F_{enc}\in R^{16\times 16\times 512}$ are concatenated to the generator. Then, $F_{dec}^{1}\in R^{32\times 32\times 256}$ , $F_{dec}^{2}\in R^{64\times 64\times 128}$ are concatenated in order. Consequently, we generated $Img_{G}\in R^{256\times 256\times 3}$ , which is a high-quality pathological ODG image. Figure 6 shows the samples from low-resolution to high-resolution ODG data generated by our network.

**Figure 7:**
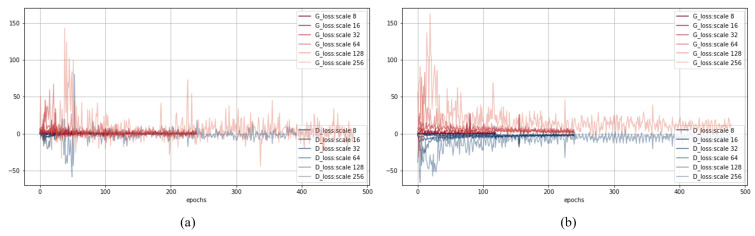
The plots of losses for all resolutions during training: (**a**) PGGAN, (**b**) proposed method.

For a more detailed description of the generation module, we used a generator and a discriminator with a symmetrical structure. In the initial generator and discriminator, 4×4 convolutional layers are used at the beginning and end, respectively, and the kernel sizes of all other convolutional layers are 3×3 . Both networks used the convolutional layer twice per scale for each progress and use LeakyReLU activation. The generator generates the RGB data $Img_{G}$ as an output using a 1×1 convolutional layer and tanh at the final stage, and the discriminator at the end applies a 4×4 convolutional layer and a dense layer to distinguish synthetic and real data. The discriminator gradually add layers while using the weights of the previous training step during the training process following the PGGAN method. The training progressed well without applying a separate training method. Figure 7 shows samples generated by the proposed method.

### 3.3. Training

In practice, applying the embedding feature concatenation method requires a pre-trained segmentation module. The segmentation network was trained using supervised learning with the softmax outputs and the final layer of the network and the k-means clustering guide set. The k-means clustering results can be considered a multi-label classification problem; categorical cross-entropy (CCE) was applied as an objective function. Therefore, the segmentation loss that was used can be formulated as:

(2) $$L_{seg}=CCE\left(S\left(x\right),c\right)\;$$

(3) c=Cluster(x)

where S(·) denotes the segmentation network, x is the input data sample from the input dataset $\left\{x_{i}|i=1,\ldots ,N\right\}$ , and c is the corresponding centroid from the cluster set $\left\{c_{i}|i=1,\ldots ,N\right\}$ , which is the result of the k-means clustering algorithm.

We used the k-means clustering guide set because our training dataset does not have corresponding ground truth labels. Unlike natural images, in which local features are important, pixel-level labels can be created based on clusters that correspond to fairly simple features in the pathological images. When the guide labels are obtained through the k-means clustering algorithm, it is not experimentally more accurate than the paired dataset with the ground truth, but it leads to reasonable segmentation performances. The model delivered good performance when K=4 or 5, and it was fixed at K=4 in the experiment.

The reconstruction network, R(·) , reconstructs the given input images x from S(x) , which are the masked samples. We applied the mean squared error (MSE) to the training to reduce the the difference between the original data input *x* and the reconstructed data R(S(x)) . Therefore, the reconstruction loss was formulated as follows:

(4) $$L_{recon}=MSE\left(R\left(S\left(x\right)\right),x\right)\;$$

By adding the reconstruction model and loss to a single segmentation model, we obtained more highly accurate segmentation results. Finally, the total loss of the segmentation module was utilized by adding $L_{seg}$ and $L_{recon}$ , as shown in Equation (5):

(5) $$L_{total}=L_{seg}+\lambda_{recon}L_{recon}$$

where $\lambda_{recon}$ is a reconstruction parameter heuristically set to 5.

We used the WGAN-GP objective function [38] as an adversarial objective function to train the generation module, and this objective function converges with that of our feature concatenation method. The GAN objective function is formulated as follows:

(6) $$L_{gan}=E_{\tilde{x}∼P_{G}}\left(\left[D\left(\tilde{x}\right)\right]\right)-E_{x∼P_{r}}\left(\left[D\left(x\right)\right]\right)+\lambda_{GP}E_{\hat{x}∼P_{\hat{x}}}([∥{\left(\nabla_{\hat{x}}D\left(\hat{x}\right)-1\right)}^{2}{∥}_{2}])\;$$

where $\tilde{x}$ denotes a synthesis output from the distribution $P_{G}$ , x denotes an image from the real distribution $P_{r}$ and $\hat{x}$ denotes a sample image from sample distribution $P_{\hat{x}}$ . We define $\hat{x}$ as the formula of $\hat{x}\leftarrow \epsilon x+\left(1-\epsilon \right)\tilde{x}$ and set the penalty coefficient $\lambda_{gp}$ to 10 as used in [38]. Figure 7 shows the losses of the discriminator and generator according to the training epochs of PGGAN and the proposed method. Each graph shows the training results from scale 8 to 256 pixels. Figure 7a plots the training curves of PGGAN and Figure 7b plots the training curves of the proposed method.

Our networks were implemented using Tensorflow and Keras. We optimized the Generation Module using Adam with $\beta_{1}$ and $\beta_{2}$ , 0.9 and 0.99 , respectively. The module generator and discriminator had the same learning rate ( 0.0001 ). We trained the networks using an Intel Core i7-10700 2.9 GHz and NVIDIA GPU RTX 3090 with 24 GB GPU memories. The module required approximately a week for training.

## 4. Experimental Results

In this section, we describe the experiments conducted to measure the performance of the proposed model in two ways. First, we compared the quality of the images generated using a qualitative evaluation method. To measure qualitative scores, we used modified AMT perceptual studies [61,62,63]. We experimented using perceptual surveys in paired and unpaired settings. Through these experiments, we compared the confusion rates of the generation results from the proposed method with the embedding feature concatenation method and other methods, WGAN-GP, PGGAN and Boyd et al. [57].

Second, to compare the two models, we calculated the Fréchet Inception Score (FID) [64] and Inception Score (IS) [65]. The score is a commonly used metric for measuring the performance of generative models. We calculated the two metrics and described the advantages of applying our method compared to the other methods.

### 4.1. Preparing Data

We used data labeled as oligodendroglioma among the primary diagnosis classifications of TCGA-LGG as training data. The dataset was collected from https://portal.gdc.cancer.gov/ (accessed on 20 December 2021). The TCGA-LGG ODG dataset consists of H&E stained pathology slides. We cropped whole-slide images to 512×512 patches based on a 40× magnification. Subsequently, we resized it to 256×256 pixels to simplify this task. We then removed the patches that were located at the boundary and that showed too little tissue because these negatively affect the training. We constructed a training dataset with a total of 120 k samples by cropping slides from the TCGA-LGG ODG dataset as patches and filtering out unnecessary data.

### 4.2. Examples of the Proposed Method

**Figure 8:**
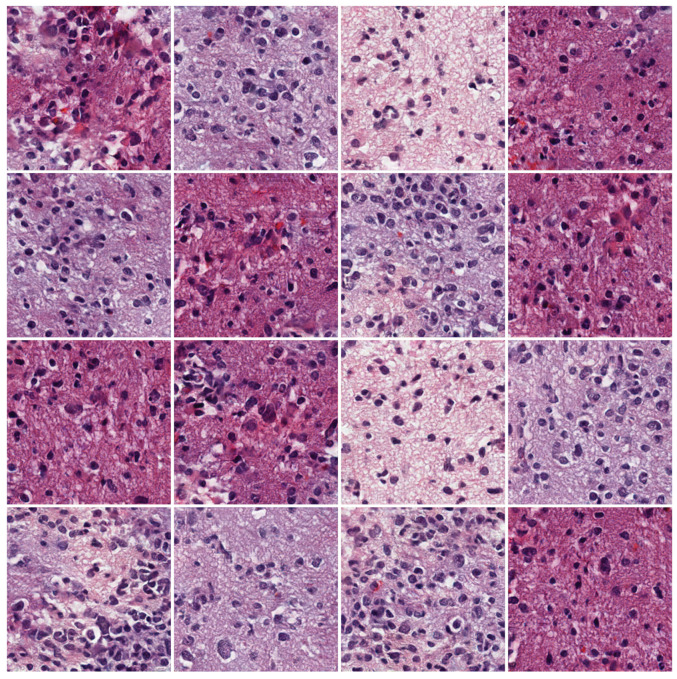
Oligodendroglioma samples with a resolution of 256×256 generated by the proposed method.

We trained our network using a pre-processed ODG dataset using synthetic ODG data with a resolution of 256×256 pixels. Figure 8 shows some of the samples generated by the proposed model. The proposed model captured the morphological features seen in pathological images such as cells, nuclei, and tissues while generating visually superior results. However, unrealistic tissue images were occasionally synthesized, indicating that the model can be improved.

**Figure 9:**
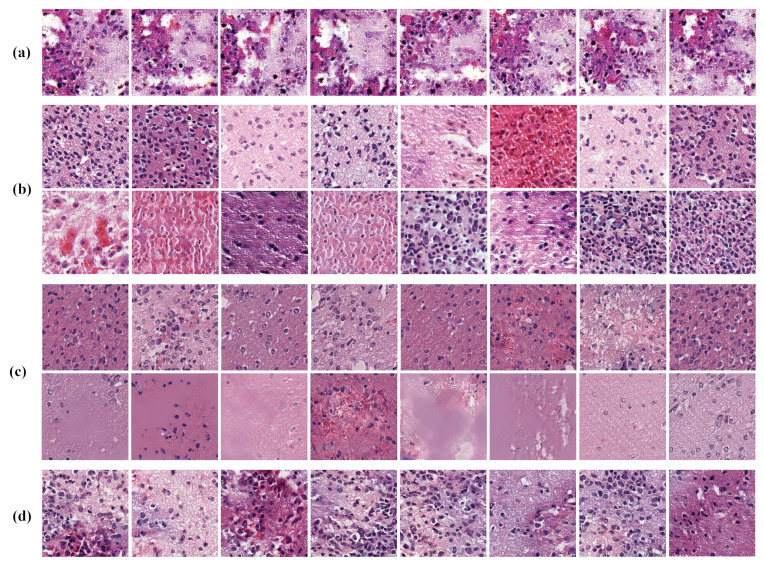
Generated samples from the WGAN-GP, PGGAN, Boyd et al. and our model. (**a**) The WGAN-GP samples generate images with a poorly uniform staining color and quality. (**b**) The PGGAN samples contain unrealistic patterns that do not exist in real data. (**c**) The samples from Boyd et al. generate images with high quality (upper row) but sometimes fail to generate (lower row). (**d**) The samples from our model generate images with a poorly uniform staining color.

Although there is a color difference between slides in the H&E-stained tissue images, the tissues are stained with the same color in each slide because of the nature of staining. However, we observed samples in which the extracellular material’s staining was not uniform in the generated patch images. As shown in Figure 9d, the color of some samples changed to a different staining color. These samples indicate a limitation of our model that must be addressed.

### 4.3. Qualitative Evaluation

We used a qualitative method to measure how distinct the synthetic ODG images we generated are from real data. We performed the perceptual experiments as a survey to measure the human confusion rate under two settings, and we designed a variation of AMT perceptual studies. The survey was conducted under two settings as follows:

1. Paired Setting: Subjects are shown sequences of real and synthetic image pairs for four seconds. Each trial required them to choose which they thought was the real image. The experiment consisted of 200 trials per subject, and the first 20 trials gave the correct answers as feedback.
2. Unpaired Setting: Subjects were randomly shown one of the WGAN-GP results, the PGGAN results, the results of Boyd et al., our results, or the real images. The subjects were asked to choose whether the images shown in each trial were either real or synthetic. This survey included a total of 250 trials, and the first 25 trials provided the correct answers as feedback.

We surveyed 72 subjects with general medical knowledge, including specialists in pathology. We configured all images used in the survey to have 256×256 resolution (but generated images by Boyd et al. were produced with 224×224 ) and calculated the confusion rate as the average of the subjects’ responses. The confusion rate of each subject means the rate at which the subjects answered labeled real among the fake images, and a larger value means that the synthetic data were judged as from real domain.

The confusion rates of the subjects are listed in Table 1. In a paired setting, the model increased the confusion rate by approximately 12.23% to 55.19 ± 11.18 higher than the PGGAN of 42.96 ± 12.76. For the unpaired setting, the confusion rate of our model was 51.25 ± 13.93, which is a 12.5% increase over the PGGAN of 38.75 ± 16.26. As shown in Figure 9c, the method of Boyd et al. also synthesized high-quality images and recorded the second highest confusion rates of approximately 52.13% and 50.89% for each setting. Compare to the method of Boyd et al., our model showed 3.06 and 0.36% higher performance for paired and unpaired settings, respectively.

### 4.4. Quantitative Evaluation Metrics

We used two metrics for the quantitative evaluation. The first is the FID score, which measures the distance between the images generated by the trained and training datasets. In addition, for comparison with our model, we measured the FID of the other methods generation results. We configured the number of data samples equal to the training data for the FID calculation and used the Inception-V3 model [66] pre-trained on ImageNet [50]. We also calculated the IS and compared its performance with that of the other methods. The IS calculates a score for the fidelity and diversity of the generated image and higher value means better performance.

The FID and IS values are listed in Table 2. Our model scored the second lowest FID score of 34.96359 , which is 4.28195 higher than the PGGAN. The lower the FID, the better the performance. This means that the features of the PGGAN samples extracted from the Inception-V3 matched the real data distribution more accurately. In the case of IS, our method measured the highest score of approximately 2.91609 higher, indicating that the fidelity and diversity were higher than that of the other methods.

## 5. Discussion

Due to the increasing interest in using deep learning for digital pathology, we conducted a study to synthesize ODG data to solve the problem of obtaining sufficient medical data.

We propose a method of concatenating the embedding features extracted from the segmentation module to the base network generator. Consequently, there was no improvement in the FID, but the IS quantitative results improved compared with the base network PGGAN. FID and IS evaluated the performance using the ImageNet-pre-trained model. Therefore, there was a difference in the domain because our dataset consisted of only pathology images. The pathology data-generation task achieved a low score compared with the general datasets in both metrics because both the base model and our generated images are from a similar domain.

Therefore, we made a subjective comparison through a qualitative evaluation. We conducted a survey, consisting of two settings as a qualitative evaluation, and our results achieved higher scores for both settings. In the paired setting, the confusion rate increased by approximately 12.23%, and in the unpaired setting, it increased by approximately 12.5%. Our results show that the confusion rates measured respectively 55.19%, 51.25% in the survey settings, indicating that the subjects could not reliably distinguish between the real and the synthetic images.

There are differences in the visual quality compared to the generation results of the PGGAN. Figure 9b shows examples of good and bad samples generated by the PGGAN. The upper row contain good samples and the lower row contain bad samples. The PGGAN synthesized high-quality pathological images; however, unnatural patterns that do not exist in the real domain are also generated. However, our proposed method created more realistic images of pathological characteristics. Our proposed method improved the visual performance by solving the problem of the baseline network that generated artifacts, i.e., strange patterns.

Although the proposed method raises the problem of normalizing the staining color, addressing it is beyond the scope of this study. However, we hope that future research will solve this problem and demonstrate the potential for pathological image generation with higher resolution. Furthermore, we think that future studies will achieve advanced performance in data augmentation and binary classification tasks on positive and negative datasets.

## 6. Conclusions

This research was intended to improve visual performance over the existing model for generating oligodendroglioma pathology data.

Unlike the existing method, the proposed method extracts embedding features with the segmentation module and uses it for training the generator. We used k-means clustering to train the segmentation module on the training dataset without labels. The reconstruction network was used to extract embedding features and concatenate them to the layers of the generator. Our generator synthesized pathological data from the linked embedding features and latent vectors.

Although the proposed method has a problem with normalizing the staining color, it consistently generated samples with high visual quality. The results were analyzed by comparison with the PGGAN used as a baseline, and low FID and high IS values were achieved. Compared to subjective evaluation by humans, the proposed method exhibited a higher confusion rate. However, the results show that our method causes more confusion in the subjective evaluation of humans. Therefore, the proposed method could visually complement the baseline network.

We expect that the proposed method will generate more realistic pathology data by solving the problem of normalizing the staining color in future studies. We hope that extended studies will synthesize meaningful data as a data augmentation technique for the binary positive and negative classification of many tumors.

## Figures and Tables

**Table 1:** The qualitative evaluation results of our study. The confusion-rate measurement result by model in the paired setting (top). The confusion-rate measurement result by model in the unpaired setting (bottom).

Survey Setting	Model	Confusion Rate (%) ↑
paired	WGAN-GP [38]	15.28 ± 12.63
	PGGAN [45]	42.96 ± 12.76
	Boyd et al. [57]	52.13 ± 10.37
	Ours	55.19 ± 11.18
unpaired	WGAN-GP [38]	13.89 ± 18.08
	PGGAN [45]	38.75 ± 16.26
	Boyd et al. [57]	50.89 ± 18.58
	Ours	51.25 ± 13.93

**Table 2:** The comparison of quantitative evaluation through FID and IS.

Model	FID ↓	IS ↑
Real Data	-	2.85245 ± 0.01389
WGAN-GP [38]	283.50172	1.45169 ± 0.01101
PGGAN [45]	30.68164	2.40684 ± 0.03606
Boyd et al. [57]	37.11364	2.73736 ± 0.02308
Ours	34.96359	2.91609 ± 0.03873

## Data Availability

Not applicable.
